# Emergence of Multidrug-Resistant *Escherichia coli* Harbouring the CS31A Virulence Factor in Neonatal Calf Diarrhoea in Central France

**DOI:** 10.3390/ani15192844

**Published:** 2025-09-29

**Authors:** Clémence Provost, Hadjila Yanes, Guillaume Mosnier, Tiago Lima, Gabriela Jorge da Silva, Ana Rita Pedro, Maria José Saavedra, Eduarda Silveira

**Affiliations:** 1 Departamento de Ciências Veterinárias, Escola Universitária Vasco da Gama, 3020-210 Coimbra, Portugal; 2 TERANA—Laboratoires D’Analyses por La Santé Publique, 20, rue Aimé Rudel, 63370 Lempdes, Puy-de Dôme, France; 3 Faculty of Pharmacy, University Coimbra, 3000-458 Coimbra, Portugal; 4 CNC-UC—Center for Neuroscience and Cell Biology, University Coimbra, 3004-517 Coimbra, Portugal; 5 CIBB—Centre for Innovative Biomedicine and Biotechnology, University Coimbra, 3004-548 Coimbra, Portugal; 6 CIVG—Centro de Investigação Vasco da Gama, 3020-210 Coimbra, Portugal; 7 Antimicrobials, Biocides & Biofilms (AB2Lab), Department of Veterinary Sciences, University of Trás-os-Montes and Alto Douro, 5000-801 Vila Real, Portugal; 8 CITAB—Inov4Agro, Centre for the Research and Technology of Agro-Environmental and Biological Sciences, University of Trás-os-Montes and Alto Douro, 5000-801 Vila Real, Portugal; 9 CECAV—AL4AnimalS, Animal and Veterinary Research Center, University of Trás-os-Montes and Alto Douro, 5000-801 Vila Real, Portugal; 10 CERNAS—Research Center for Natural Resources, Environment and Society, 3045-601 Coimbra, Portugal

**Keywords:** Neonatal Calf Diarrhoea, *Escherichia coli*, virulence factors, adhesins, multidrug-resistance, antimicrobials

## Abstract

*Escherichia coli* is a significant cause of Neonatal Calf Diarrhoea (NCD). Its extensive antigenic diversity, coupled with the ability to acquire antimicrobial resistance determinants, hampers treatment effectiveness and compromises the control measures. This study investigated the link between the presence of multidrug-resistant (MDR) *E. coli* and virulence factors (VFs) in NCD from central France (Departments of Cantal, Haute-Loire, Loire, and Puy-de-Dôme), between 2016 and 2022. *E. coli* was identified at TERANA Laboratories, France, using API 20E (BioMérieux^®^) and MALDI-TOF Mass Spectrometry. Virulence factors, namely adhesins, were assessed with the slide agglutination method, and antimicrobial susceptibility testing was conducted across various antimicrobial classes. Out of 2367 *E. coli* strains isolated from cases of NCD, a high percentage were resistant to aminopenicillins (88.8%), aminoglycosides (89.1%), tetracyclines (79.7%), quinolones (48.4%), and sulphonamides (42.4%). More than half (58.6%) carried VFs, and 84.9% exhibited MDR profile, of which 61.34% (1233/2010) also harboured VFs. The adhesin CS31A-producing *E. coli* was the most prevalent, followed by the fimbrial adhesins F5 and F17 (60.8%, 20.0%, and 8.3%, respectively), all of which were associated with a high prevalence of MDR strains (79.1–93.9%). The highest occurrence of MDR profiles was observed in *E. coli* strains carrying CS31A and in those lacking VFs, both groups showing co-resistance to aminopenicillins, aminoglycosides, and tetracyclines or sulphonamides. The calf production sector may act as a reservoir for MDR *E. coli* strains, regardless of the presence of VFs, posing a major threat to public health and safety.

## Simple Summary

Enterotoxigenic *Escherichia coli* (ETEC) exhibits a high prevalence in Neonatal Calf Diarrhoea, leading to significant economic losses in the animal production sector. This study aimed to correlate the presence of virulence factor-producing multidrug-resistant *E. coli* in central France, specifically in the departments of Cantal, Haute-Loire, Loire, and Puy-de-Dôme. A large number of diarrhoeal stool samples from calves were analysed at TERANA Laboratories (France), revealing a high rate of multidrug-resistant *E. coli*, which harbour different virulence factors, namely fimbrial and non-fimbrial adhesins. Among *E. coli* strains carrying adhesins, a high prevalence of multidrug-resistance was observed, averaging 84.9%, with a predominance of CS31A-producing strains. These findings underscore the importance of monitoring the co-occurence of adhesins and multidrug-resistant profiles in *E. coli* from Neonatal Calf Diarrhoea, as they could serve as a reservoir for strains harbouring both determinants, thereby posing a significant public health risk. To our knowledge, this study represents the first large-scale report on the correlation between multidrug resistance profiles and virulence factors-producing *E. coli* isolated from cases of Neonatal Calf Diarrhoea.

## 1. Introduction

Neonatal Calf Diarrhoea (NCD) is a leading cause of morbidity and mortality among calves under one month old, resulting in significant economic losses in dairy and beef cattle production [1]. NCD is a highly complex condition that can be caused by bacterial, viral, or parasitic pathogens, with co-infections frequently reported [1,2,3,4].

*E. coli*, a Gram-negative bacillus belonging to the order *Enterobacterales*, is highly prevalent in cases of NCD [3]. Although *E. coli* is part of the gastrointestinal microbiota in both humans and animals [5,6,7], it can become pathogenic when the intestinal barrier is compromised, leading to translocation into the abdominal cavity and potentially resulting in peritonitis or septicaemia [5,8]. Conversely, pathogenic strains of *E. coli* encode specific virulence factors (VFs) responsible for various infectious diseases collectively known as colibacillosis [7,8]. Pathogenic *E. coli* is classified into two groups: Extra-intestinal *E. coli* (ExPEC) and Intestinal *E. coli* (InPEC) [8,9]. Enterotoxigenic *E. coli* (ETEC), an InPEC pathotype, is a primary pathogen recognised as the principal bacterial agent causing NCD [1,3,10].

The initial step of the virulence mechanism of ETEC involves its adhesion to the intestinal epithelium via fimbrial and non-fimbrial adhesins [11,12]. The fimbrial adhesins implicated in this process include F5, F17, and F41 [10]. Additionally, the non-fimbrial adhesin CS31A is a surface antigen immunologically related to fimbriae F4 and F41. CS31A is an important virulence factor of *E. coli*, involved not only in NCD but also associated with septicaemia, particularly in young animals [10,11].

The management of NCD often involves the use of diverse antimicrobials, including beta-lactams, aminoglycosides, quinolones (including fluoroquinolones), phenicols, tetracyclines, polymyxins, and sulphonamides [13]. Some of these antibiotic classes are classified as critically important for human medicine [14,15]. A high prevalence of *E. coli* carrying various antimicrobial resistance (AMR) mechanisms has been documented in both humans and animals, including those from food-producing settings. This raises significant concerns regarding the implications of antimicrobial use in livestock and the potential transmission of AMR bacteria to humans through the food chain [6,7,16,17,18,19,20,21]. However, large-scale data on the association between distinct MDR profiles and VFs-producing *E. coli* in NCD remain limited.

The main objective of this study was to investigate potential associations between multidrug resistance (MDR) profiles and specific VFs, particularly adhesins, in *E. coli* isolates from cases of NCD. Unlike surveillance or risk assessment studies, our focus was on correlation analysis, aiming to identify potential biological links between AMR and adhesin patterns. To this end, we conducted a retrospective study including *E. coli* strains collected between 2016 and 2022 from departments in central France (Cantal, Haute-Loire, Loire, and Puy-de-Dôme), with analysis of both AMR and adhesin profiles.

## 2. Materials and Methods

### 2.1. Database Source and Management

The clinical data used in this study were provided by TERANA Laboratories (France), which operates across 10 departments in the country: Cantal, Cher, Creuse, Drôme, Indre, Loire, Haute-Loire, Nièvre, Puy-de-Dôme, and Rhône.

The data set analysed in this study included information on clinical samples (animal species, date of sample collection, and origin) and bacteriological results (bacterial identification, presence of virulence factors, and antimicrobial susceptibility testing results). Duplicate samples and repeated results (defined as isolates with the same date and reference) were excluded. Only one isolate per calf per submission was retained in the data set, ensuring that each isolate represented a unique sampling event. In addition, data containing confidential information were removed in accordance with Data Protection Law [22].

### 2.2. Study Design and Sample Characterisation

Between 2016 and 2022, TERANA Laboratories (https://www.labo-terana.fr/ accessed on 4 January 2023) received 2367 diarrhoeic fecal samples from young cattle diagnosed with NCD. These samples were collected during routine clinical practice in the Departments of Cantal, Haute-Loire, Loire, and Puy-de-Dôme in central France.

### 2.3. Identification and Virulence Profiling of Bovine Diarrheagenic Isolates

Identification of *E. coli* was performed using API 20E (BioMérieux^®^, Marcy-l’Étoile, France) for the period between 2016 and 2019 and MALDI-TOF MS (Matrix-assisted laser desorption/ionization time-of-flight mass spectrometry) (Bruker Daltonics, Bremen, Germany) from 2020 onwards. All isolates were subsequently screened for fimbrial (F5, F6, F17, F41) and non-fimbrial (CS31A) VFs (virulence factors, namely bacterial adhesins) using the slide agglutination method [23].

### 2.4. Antimicrobial Susceptibility Analysis

Antimicrobial Susceptibility Testing (AST) was performed using the disk diffusion method or determination of the Minimum Inhibitory Concentration (MIC), following the guidelines of the *Comité de l’Antibiogramme de la Société Française de Microbiologie* (CA-SFM) and the European Committee on Antimicrobial Susceptibility Testing (EUCAST), according to standard NF U47-107 [23]. A total of 17 antimicrobials from various classes were evaluated, including beta-lactams with or without beta-lactamase inhibitors namely amoxicillin (AML), amoxicillin-clavulanic acid (AMC); cephalexin (CL), cefoxitin (FOX), cefuroxime (CXM), ceftiofur (EFT), and cefquinome (CEQ); the aminoglycosides—gentamicin (CN), neomycin (N), and streptomycin (S); the quinolones—nalidixic acid (NA), flumequine (UB), and marbofloxacin (MBF); the phenicols—florfenicol (FFC); the tetracyclines—tetracycline (T); the polymyxins—colistin (C); and sulfamethoxazole-trimethoprim (SXT).

Susceptibility testing to colistin was performed using the Colispot method developed by *Agence Nationale Sécurité Sanitaire de l’ Alimentation, de l’Environnement et du Travail* (ANSES) [24,25].

The isolates exhibiting resistance to three or more classes of antimicrobials were considered MDR, by established definitions [26].

### 2.5. Statistical Analysis

The prevalence of AMR and VFs-producing *E. coli*, as well as the association between these determinants and their MDR profiles, was statistically evaluated using the Chi-square test. Statistical significance was set at *p* < 0.05. All analyses were performed using GraphPad Prism^®^ (version 9.4.1).

### 2.6. Ethical Approval

This study was reviewed and approved by the Ethics Committee of Escola Universitária Vasco da Gama, Coimbra, Portugal, under the internal reference number 20/2024.

## 3. Results

### 3.1. Adhesins Identification

A total of 2367 *E. coli* isolates were identified, comprising various serotypes. Among these, 58.6% (1388/2367) carried fimbrial and non-fimbrial adhesins, while 41.4% (979/2367) carried none (*p* < 0.05).

The adhesin CS31A was the most prevalent VF. It was identified in 60.8% (844/1388) of isolates harbouring VFs, a significantly higher proportion compared to all other VFs detected among the *E. coli* isolates analysed (*p* < 0.05).

In addition, 6.8% (94/1388) of the isolates harboured more than one adhesin concurrently (Table 1).

### 3.2. Frequency of Antimicrobial Resistance

The AST of *E. coli* isolates obtained from NCD cases did not consistently include all antimicrobial classes, as this depended on the antimicrobial panels requested by the submitting veterinarian. Consequently, variation was observed in the number of *E. coli* isolates tested for each antimicrobial agent.

The results of the AST among *E. coli* isolates from NCD, collected during the study period revealed a high prevalence of resistance to aminopenicillins and aminoglycosides (amoxicillin: 88.8%, 1646/1854; streptomycin: 89.1%, 1776/1994), followed by tetracyclines (tetracycline: 79.7%, 1856/2330), quinolones (nalidixic acid: 48.4%, 435/899), and sulphonamides (sulfamethoxazole-trimethoprim: 42.4%, 1003/2363) (*p* < 0.05).

**Figure 1:**
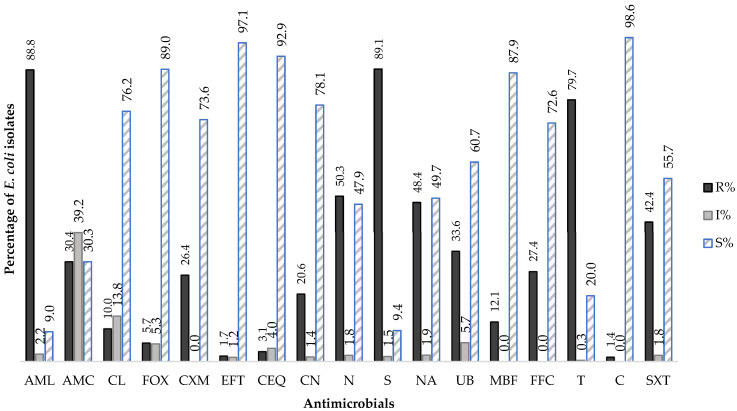
Global distribution average of Antimicrobial Susceptibility profile of *E. coli* isolated from NDC cases (2016–2022). R—resistant; I—susceptible to increase exposure; S—susceptible; AML—amoxicillin, AMC—amoxicillin with clavulanic acid, CL—cephalexin, FOX—cefoxitin, CXM—cefuroxime, EFT—ceftiofur, CEQ—cefquinome, CN—gentamicin, N—neomycin, S—streptomycin, NA—nalidixic acid, UB—flumequine, MBF—marbofloxacin, FFC—florfenicol, T—tetracycline, C—colistin, SXT—sulphamethoxazole with trimethoprim.

Furthermore, 30.4% (718/2360) of *E. coli* isolates were resistant to amoxicillin with clavulanic acid, while 39.2% (926/2360) exhibited susceptibility to increased exposure, following the EUCAST redefinition of “Intermediate Susceptibility” introduced in 2019. Among cephalosporins, resistance to cefuroxime was the most notable (26.4%, 396/1502), followed by cephalexin (10.0%, 186/1863), cefoxitin (5.7%, 95/1674), cefquinome (3.1%, 72/2352), and ceftiofur (1.7%, 40/2339) (*p* < 0.05). Regarding fluoroquinolones, a non-significant resistance rate of 33.6% (237/705) was detected for flumequine (*p* > 0.05), in contrast to a markedly lower rate recorded for marbofloxacin at 12.1% (286/2363) (*p* < 0.05). Resistance to colistin remained low, at 1.4% (31/2233) (Figure 1; Table 2).

Regarding the evolution of *E. coli* resistance profiles to the antimicrobials tested over the study period (2016–2022), the results revealed significant fluctuations in resistance to amoxicillin-clavulanic acid, cefquinome, gentamicin, cefuroxime, ceftiofur, florfenicol, cefoxitin, marbofloxacin, nalidixic acid, and tetracycline (*p* < 0.05). Until 2019, a progressive increase was observed in the number of *E. coli* isolates resistant to amoxicillin-clavulanic acid, cefquinome, gentamicin, streptomycin, florfenicol, and sulphamethoxazole-trimethoprim, followed by a significant decrease in 2020 (*p* < 0.05). A significant upward trend in cefuroxime resistance was observed, rising from 13% to 31% (*p* < 0.05). In contrast, cefquinome and ceftiofur (both fourth-generation cephalosporins), as well as marbofloxacin (a fluoroquinolone), showed significant downward trends in resistance (*p* < 0.05).

It is also noteworthy that the results indicate low rates of susceptible to increased exposure to antimicrobials, except for the combination of amoxicillin and clavulanic acid, for which a steady increase was recorded, rising from 25% (67/268) in 2016 to 47.4% (170/358) in 2022 (*p* < 0.05) (Table 3). Additionally, the resistance rate to amoxicillin was decreased when used in combination with clavulanic acid (Figure 1).

### 3.3. Adhesins Co-Carriage and Multidrug-Resistance in E. coli Isolated from Cases of Neonatal Calf Diarrhoea

The findings revealed that 84.9% (2010/2367) of the *E. coli* isolates collected during the study period exhibited an MDR profile (*p* < 0.05). The prevalence of MDR strains remained statistically stable over time, with no significant year-to-year variation detected (*p* > 0.05) (Table 4). Although not statistically significant, a noticeable increase in MDR prevalence was recorded in 2022 (21.9%) compared to 2016 (10.6%) (Table 4).

**Figure 2:**
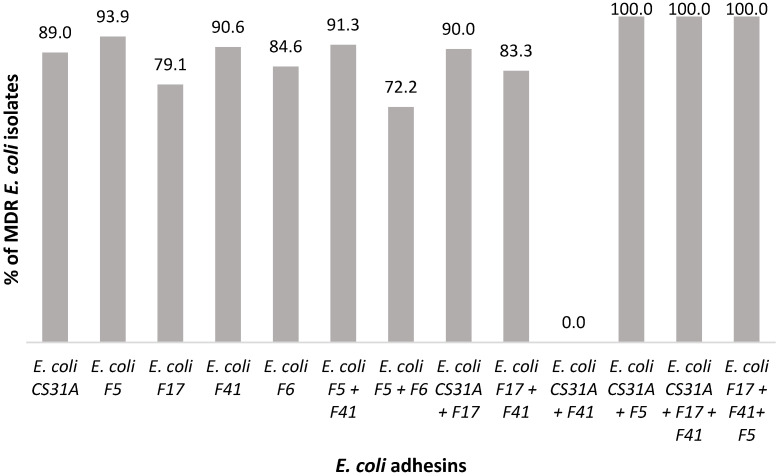
Correlation between *E. coli* pathotypes carrying adhesins and MDR profile from NCD cases (2016–2022).

It is noteworthy that more than half of the MDR *E. coli* isolates (61.2%; 1230/2010) harboured the adhesins tested, and 88.6% (1230/1382) of the characterised pathotypes exhibited an MDR profile. Notably, the pathotypes *E. coli* CS31A (37.3%; 751/2010) and *E. coli* F5 (12.9%; 260/2010) displayed significantly higher occurrences of MDR, whereas other pathotypes showed no significant deviation (*p* < 0.05; *p* > 0.05, respectively) (Figure 2; Table 5). Nonetheless, when assessing the association between different *E. coli* pathotypes and the presence or absence of MDR profiles, these findings demonstrated a statistically significant risk of being associated with MDR (*p* > 0.05).

## 4. Discussion

Despite the extensive scientific literature on AMR in *E. coli*, there remains a notable lack of studies specifically addressing the role of defined pathovar-associated virulence factors in NCD. This study addresses that gap by focusing on *E. coli* strains isolated from NCD cases, demonstrating a high prevalence of AMR among calves in central France, including MDR profiles associated with strains carrying CS31A or F5.

Whenever possible, official data published by *Réseau de d’épidémiosurveillance de l’antibiorésistance des bactéries pathogènes animales* (RESAPATH) were used as a reference to assess whether the findings of this study align with the national data and trends.

The high prevalence of CS31A or F5-producing strains observed in this study is consistent with previous reports. Indeed, F5-producing *E. coli* has frequently been associated with NCD [2,12], while other studies have reported a higher prevalence of the CS31A [6]. Notably, CS31A has been linked not only to clinical cases of diarrhoea but also to septicaemia in young animals [11]. Although CS31A-producing strains displayed the highest prevalence of MDR in our data set, no adhesin, including CS31A, was statistically associated with an increased risk of MDR. The observed link should therefore be interpreted as a descriptive finding rather than a statistically proven risk factor.

Regarding the distribution of *E. coli* AMR patterns in animals with NCD, our results do not consistently match with official data, particularly for fluoroquinolones. For instance, the prevalence of resistance to marbofloxacin in this study (12%) is notably higher than the 5–7% reported in RESAPATH data [27]. Additionally, between 2018 and 2022, France recorded a national increase in the proportion of *E. coli* isolates classified as susceptible to increase exposure to amoxicillin with or without clavulanic acid. This trend is consistent with the findings of our study, which recorded an even higher prevalence of such isolates in central France [27]. It is also worth noting that the observed resistance rates to colistin, tetracycline, and quinolones in this study were broadly in agreement with the official data reported [27].

Nevertheless, RESAPATH has highlighted the potential underestimation of colistin resistance due to the limited reliability of the disc diffusion method routinely used in France, as well as the lack of routine microdilution for MIC determination [28]. It is therefore plausible that the colistin resistance rate observed in this study (1.4%) was similarly underestimated. Even so, the result remains consistent with national data, which reports resistance levels below 2% in *E. coli* from NCD cases [28].

According to official French data, there has been a documented decrease in the prevalence of MDR strains among *E. coli* isolates from animals with NCD between 2012 and 2022 [27]. However, our study identified an increase in the prevalence of MDR *E. coli*, rising from 10.6% in 2016 to 15.4% in 2022, with a peak of 21.9% in 2021. In this study, the most prevalent MDR profile was characterised by resistance to amoxicillin, gentamicin, tetracycline, and sulfamethoxazole-trimethoprim or nalidixic acid, closely mirroring trends observed in official data. This pattern may be attributed to the widespread use of these antimicrobials in animal production settings [29]. Furthermore, across Europe, this resistance profile has emerged as the most prevalent, with high or very high resistance rates reported in all animal categories. Notably, *E. coli* isolates from cattle under one year of age have shown resistance to ampicillin, sulfamethoxazole, trimethoprim, and tetracycline [20].

These findings are particularly concerning given the overlap in MDR patterns observed in *E. coli* from both healthy animals and those affected by NCD, suggesting a possible role for horizontal gene transfer events in disseminating resistance genetic elements, and potentially encoded plasmids adhesins like-CS31A across *E. coli* populations [30].

To explore a potential link between *E. coli* isolated from NCD cases and human infections, data from the European Antimicrobial Resistance Surveillance Network (EARS-Net) for 2021 indicated that approximately 52.3% of human *E. coli* isolates were resistant to at least one antimicrobial class, namely aminopenicillins, fluoroquinolones, third-generation cephalosporins, aminoglycosides, and carbapenems. Resistance was highest to aminopenicillins (53.1%), followed by fluoroquinolones (21.9%), third-generation cephalosporins (13.8%), and aminoglycosides (9.6%) [31]. As supported by other previous studies, high levels of aminopenicillin resistance have been reported in both humans and animals, including exotic pets [20,31,32,33].

Altogether, a comparative analysis of the results from this study and previously published data reveals a consistent pattern across different ecological niches, including humans and animals. This pattern warrants further research to inform strategies aimed at containing the spread of *E. coli* strains with MDR profiles and VFs. Ongoing surveillance of AMR and virulence determinants in other species, including humans, livestock, and companion animals, is crucial to pre-emptively address emerging public health threats.

Although this study provides valuable insights into the relationship between virulence factors and MDR profiles in *E. coli* strains from NCD, it is limited by the absence of molecular analyses of resistance mechanisms and genes, as only phenotypic data from the French public laboratory network TERANA were available. Even so, the findings highlight the calf production sector as a potential reservoir of MDR strains and emphasise the need for continued surveillance and future studies incorporating molecular approaches.

Additionally, as the study is based on diagnostic laboratory submissions, a potential selection bias must be acknowledged. The analysed samples originated from calves with clinical NCD for which veterinarians requested bacteriological testing, which means our data set is likely enriched in more severe or complicated cases. Consequently, the results may not fully reflect the overall epidemiology of *E. coli* in the general calf population. Nevertheless, given the large number of isolates analysed over a six-year period, our findings provide robust evidence of MDR–virulence associations in clinically relevant *E. coli* strains.

Importantly, France is currently implementing the third iteration of its National Action Plan on Antimicrobial Resistance, *Le plan Écoantibio 3* (2023–2028), which aims to further reduce antimicrobial exposure across various animal species and sectors. This initiative has already contributed to a 52% reduction in antimicrobial use, addressing international concerns and complying with European regulations.

## 5. Conclusions

Our data reveal an alarmingly high prevalence of MDR among *E. coli* isolates from NCD in central France, with frequent co-resistance to antimicrobials that are critically important for human medicine. 

MDR was common among CS31A-producing strains, highlighting a concerning convergence between this adhesin and resistance traits. Although no statistical association was detected between adhesin type and MDR, the consistent presence of MDR in CS31A-producing strains suggests an important epidemiological role, potentially driven by horizontal gene transfer.

Further research is essential to clarify these relationships and to support the implementation of effective control and prevention measures. Veterinarians have a pivotal role in implementing official recommendations and ensuring the responsible use of antimicrobials.

These findings point to regional hotspots for the emergence of MDR *E. coli* and underscore the need for targeted surveillance and robust antimicrobial stewardship.

Future studies incorporating molecular approaches will be crucial to better characterise the underlying AMR mechanisms and their epidemiological drivers.

## Figures and Tables

**Table 1:** Occurrence of fimbrial and non-fimbrial adhesins among *E. coli* strains isolated from NCD cases between 2016 and 2022.

Adhesins	Isolates (*n*)	Isolates (%)
*E. coli* CS31A	844	60.8%
*E. coli* F5	277	20.0%
*E. coli* F17	115	8.3%
*E. coli* F5 + F41	46	3.3%
*E. coli* F41	32	2.3%
*E. coli* F6	26	1.9%
*E. coli* F5 + F6	18	1.3%
*E. coli* CS31A + F17	10	0.7%
*E. coli* CS31A + F41	6	0.4%
*E. coli* CS31A + F5	6	0.4%
*E. coli* F17 + F41	6	0.4%
*E. coli* CS31A + F17 + F41	1	0.1%
*E. coli* F17 + F41+ F5	1	0.1%
Total	1388	100.0%

*n*—number of isolates; %—percentage.

**Table 2:** Antimicrobial resistance profile of *E. coli* isolated from NCD cases annually, over a six-year (2016–2022).

Year	Antimicrobials																
	AML	AMC	CL	FOX	CXM	EFT	CEQ	CN	N	S	NA	UB	MBF	FFC	T	C	SXT
2016	185/208	61/268	15/221	2/206	23/176	13/256	20/269	53/269	11/29	219/251	81/193	N.D.	42/268	51/252	176/234	1/230	101/267
2107	171/191	95/322	25/192	9/191	18/131	8/321	10/321	64/321	37/61	287/322	60/124	22/67	52/321	77/322	268/321	9/309	127/320
2018	218/243	137/383	27/242	19/242	28/158	6/384	13/384	95/384	43/84	330/366	163/235	55/140	59/384	118/384	311/384	6/370	175/383
2019	76/83	90/208	13/85	4/65	17/65	7/208	19/208	71/211	N.D.	177/193	9/17	14/47	43/211	80/210	186/209	4/196	102/211
2020	232/258	91/309	19/255	11/192	56/192	0/307	1/307	59/311	29/69	208/242	30/69	33/123	32/311	86/310	237/311	2/299	126/311
2021	444/512	131/512	55/512	36/507	169/509	4/512	7/512	78/506	N.D.	321/356	60/167	113/328	33/512	132/512	393/512	4/492	217/512
2022	320/359	113/358	32/356	14/271	85/271	2/351	2/351	66/359	45/85	234/264	32/94	N.D.	25/356	100/358	285/359	5/337	155/359

AML—amoxicillin, AMC—amoxicillin with clavulanic acid, CL—cephalexin, FOX—cefoxitin, CXM—cefuroxime, EFT—ceftiofur, CEQ—cefquinome, CN—gentamicin, N—neomycin, S—streptomycin, NA—nalidixic acid, UB—flumequine, MBF—marbofloxacin, FFC—florfenicol, T—tetracycline, C—colistin, SXT—sulphamethoxazole-trimethoprim; N.D.—not determined.

**Table 3:** *E. coli* isolates with a ‘susceptible to increased exposure’ profile from NDC cases reported annually over the study period (2016–2022).

Year	Antimicrobials																
	AML	AMC	CL	FOX	CXM	EFT	CEQ	CN	N	S	NA	UB	MBF	FFC	T	C	SXT
2016	2/208	67/268	22/221	6/206	0/176	5/256	16/269	3/269	1/29	3/251	8/193	N.D.	0/268	0/252	0/234	0/230	0/267
2107	0/191	93/322	11/192	17/191	0/131	6/321	18/321	6/321	0/61	7/322	3/124	2/67	0/321	0/322	0/321	0/309	10/320
2018	0/243	122/383	21/242	12/242	0/158	3/384	14/384	7/384	0/84	4/366	2/235	7/140	0/384	0/384	4/384	0/370	12/383
2019	0/83	66/208	10/85	5/65	0/65	4/208	8/208	2/211	N.D.	2/193	0/17	3/47	0/211	0/210	0/209	0/196	12/211
2020	5/258	117/309	30/255	7/192	0/192	3/307	9/307	6/311	3/69	2/242	0/69	10/123	0/311	0/310	4/311	0/299	6/311
2021	20/512	291/512	106/512	25/507	0/509	5/512	15/512	5/506	N.D.	4/356	2/167	17/328	0/512	0/512	0/512	0/492	0/512
2022	14/359	170/358	57/356	17/271	0/271	3/351	14/351	3/359	2/85	8/264	2/94	N.D.	0/356	0/358	0/359	0/337	3/359

AML—amoxicillin, AMC—amoxicillin with clavulanic acid, CL—cephalexin, FOX—cefoxitin, CXM—cefuroxime, EFT—ceftiofur, CEQ—cefquinome, CN—gentamicin, N—neomycin, S—streptomycin, NA—nalidixic acid, UB—flumequine, MBF—marbofloxacin, FFC—florfenicol, T—tetracycline, C—colistin, SXT—sulphamethoxazole-trimethoprim; N.D.—not determined.

**Table 4:** Evolution of Multidrug-Resistant *E. coli* strains from NCD cases (2016–2022).

Year	Total of Isolates	MDR Strains	
	(*n*)	(*n*)	(%)
2016	269	213	10.6
2017	322	278	13.8
2018	384	331	16.4
2019	211	183	9.1
2020	311	256	12.7
2021	512	440	21.9
2022	358	309	15.4
Total	2367	2010	84.9

*n*—number of isolates; %—percentage; MDR—multidrug-resistant.

**Table 5:** Distribution of MDR *E. coli* pathotypes from cases of NCD (2016–2022).

Strains	(*n*)	MDR (*n*)	MDR (%)
*E. coli* CS31A	844	751	37.36
*E. coli* F5	277	260	12.94
*E. coli* F17	115	91	4.53
*E. coli* F41	32	29	1.44
*E. coli* F6	26	22	1.09
*E. coli* F5/F41	46	42	2.09
*E. coli* F5/F6	18	13	0.65
*E. coli* CS31A/F17	10	9	0.45
*E. coli* F17/F41	6	5	0.25
*E. coli* CS31A/F5	6	6	0.30
*E. coli* CS31A/F17/F41	1	1	0.05
*E. coli* F17/F41/F5	1	1	0.05
Total	1382	1230	61.19

*n*—number of isolates; MDR—multidrug resistant; %—percentage.

## Data Availability

Data are available from the corresponding author under reasonable request.
